# A case of acute promyelocytic leukemia complicated by mitochondrial disease

**DOI:** 10.1007/s12185-025-03992-4

**Published:** 2025-05-01

**Authors:** Yukari Sakurai, Masakatsu Yanagimachi, Mieko Ito, Ayana Hirose, Naoyuki Miyagawa, Dai Keino, Tomoko Yokosuka, Fuminori Iwasaki, Satoshi Hamanoue, Masae Shiomi, Shoko Goto, Tomohide Goto, Hiroaki Goto

**Affiliations:** 1 Division of Hematology/Oncology, Kanagawa Children’s Medical Center, 138-4 Mutsukawa-2 Chome, Minami-Ku, Yokohama, Kanagawa 232-8555 Japan; 2 Division of Pediatric Neurology, Kanagawa Children’s Medical Center, Yokohama, Japan

**Keywords:** Mitochondrial disease, Reactive oxygen species, Acute promyelocytic leukemia, Anthracycline, Oxidative stress

## Abstract

A 15-year-old boy with congenital mitochondrial disease was diagnosed with acute promyelocytic leukemia. He was treated with all-trans retinoic acid, and his anthracycline dose was reduced in response to his underlying condition. He successfully achieved molecular remission and maintained this state for 4 years. In vitro drug sensitivity testing in peripheral mononuclear cells suggests that samples from patients in remission show higher sensitivity to various anticancer drugs than samples from healthy volunteers. Reduced-dose chemotherapy could be a valid treatment option for patients with mitochondrial diseases because exposure to elevated oxidative stress may contribute to increased drug sensitivity in these patients.

**Supplementary Information:**

The online version contains supplementary material available at 10.1007/s12185-025-03992-4.

## Introduction

Mitochondria are subcellular organelles that regulate a wide variety of cellular functions [1, 2]. Their main function is energy production, which involves the tricarboxylic acid (TCA) cycle and an electron transfer system, where reactive oxygen species (ROS) are generated [1]. Antioxidant defense mechanisms, including superoxide dismutase, catalase, and glutathione peroxidase minimize ROS-induced oxidative stress [1]. Mitochondrial encephalomyopathy with lactic acidosis and stroke-like episode syndrome (MELAS) is a metabolic disease characterized by mitochondrial respiratory chain dysfunction and energy deficiency in multiple organs, resulting in enhanced glycolytic pathway activity and increased ROS production [3]. Owing to a lack of energy and increased oxidative stress, patients with MELAS present with various symptoms, including neurological symptoms, growth disorders, liver dysfunction, cardiomyopathy, muscle weakness, diabetes, and hearing loss.

ROS have also been implicated in the efficacy of some anticancer drugs and can act as signaling molecules that regulate multiple biological and physiological processes, including tumor growth and metastasis [4–6]. Increased oxidative stress in patients with MELAS can affect the course of cancer treatment; however, few reports have detailed anticancer therapies for patients with MELAS [7–9]. Here, we report a case of MELAS in a patient who developed acute promyelocytic leukemia (APL) which was successfully treated with reduced-dose chemotherapy. An in vitro study using peripheral mononuclear cells obtained from the patient during leukemia remission suggested an unusually high sensitivity to various drugs.

## Patient history

A boy was diagnosed with MELAS at the age of nine. His two elder sisters were genetically diagnosed with MELAS; one of them died of heart failure at the age of three. The patient’s mother had never undergone genetic analysis; however, she was diagnosed with diabetes and hearing loss. The patient underwent a genetic analysis owing to the short stature (−3.2 standard deviations for his age) and mental retardation; the analysis revealed a mutation similar to that found in his sisters (MT-DNA A3243G).

The patient, at the age of 15, was hospitalized with a fever that lasted for three days. At hospitalization, a complete blood count test revealed pancytopenia; 6.4 g/dL of hemoglobin, 16 × 10^3^/µL platelet count, and 300/μL white blood cells with 18% neutrophils (Table 1). Disseminated intravascular coagulation and lactic acidosis were observed. Bone marrow aspiration revealed promyelocytes (62.4%), including Auer bodies and faggot cells. Promyelocytic leukemia cells with promyelocytic leukemia gene and retinoic acid receptor alpha gene (*PML*::*RARA*) fusion were noted in his peripheral blood and bone marrow specimens, leading to the diagnosis of APL. Because the patient developed bacteremia, antibiotic therapy and oral administration of all-trans retinoic acid (ATRA) were initiated.

**Table 1:** The result of laboratory examination at diagnosis

WBC	300/µL
Seg	15%
Stab	3%
Lymp	65%
Promyelocyte	14%
RBC	175 × 10^4^/µL
Hb	6.4 g/dL
Plt	1.6 × 10^4^/µL
Ret	30‰
PTINR	1.44
APTT	37.9 s
Fib	569 mg/dL
D-d	12.04 µg/mL
TAT	9.2 ng/ml
PIC	0.9 µg/mL
TP	5.5 g/dL
ALB	3.2 g/dL
T-Bil	0.9 mg/dL
AST	27 U/L
ALT	14 U/L
LDH	240 U/L
BUN	22.5 mg/dL
Cre	0.86 mg/dL
Na	133 mEq/L
K	3.8 mEq/L
Cl	101 mEq/L
Ca	8.3 mg/dL
IP	2.5 mg/dL
CRP	22.13 mg/dL
Ferr	1207.0 ng/mL
Fe	34 µg/dL
UIBC	129 µg/dL
Lactate	54.0 mg/dL
Pyruvate	4.03 mg/dL
pH	7.436
PCO	27.1 mmHg
PO	252.6 mmHg
HCO3	18.0 mmol/L
BE	−5.2 mmol/L
Blood culture: MSSA(+)	

The patient’s condition improved after treatment initiation. As ultrasonography and echocardiography showed normal heart function, daunorubicin (45 mg/m^2^/day for 3 days) and cytarabine (CA; 200 mg/m^2^/day for 7 days) were added starting on day 8 of ATRA. Dexamethasone was used to treat the differentiation syndrome from days 9 to 31. Hematological and molecular remission was achieved after 35 days of induction therapy without bleeding episodes or cardiac events. Subsequently, consolidation therapy was administered according to the Japanese Pediatric Leukemia/Lymphoma Study GroupAML-99 M3 protocol [10], excluding anthracyclines, which have a significant risk of cardiotoxicity associated with oxidative stress [11]. The total anthracycline dose of approximately 400 mg/m^2^, which was the doxorubicin equivalent in the original protocol, was reduced to 141 mg/m^2^ [12, 13]. Although infectious complications, pulmonary aspergillosis, and sepsis were observed, seven courses of chemotherapy were completed without any therapeutic changes except for anthracycline. Triple intrathecal therapy consisting of methotrexate, hydrocortisone, and CA was administered seven times. Magnetic resonance imaging performed before maintenance therapy revealed asymptomatic leukoencephalopathy. The patient received maintenance therapy with oral ATRA for two weeks every three months for one year. Two years after the completion of maintenance therapy, the leukemia remained in molecular remission and there was no evidence of cardiac dysfunction.

After the patient had achieved remission, we performed an in vitro drug sensitivity test using normal mononuclear cells (MNCs) from the patient as described previously [14, 15]. It revealed significant differences between the patient and healthy controls in terms of sensitivity to mitoxantrone, an anthraquinolone that causes oxidative stress similar to anthracycline (Supplemental Fig. 1). Moreover, the sensitivity of the patient’s MNCs to CA tended to be higher than the control counterparts. The test details are shown in the supplemental methods. Drugs of the panel, their final concentrations, and the average drug effective scores have been listed in Supplemental Tables 1 and 2.

## Discussion

MELAS is one of the most common maternally inherited mitochondrial disorders, leading to energy deficiency and increased oxidative stress associated with various organ disorders [3]. Recently, the relationship between anticancer drugs and oxidative stress has attracted attention [7, 11, 16–19]. Some anticancer drugs enhance oxidative stress in both normal and cancer tissues. This has led to the hypothesis that patients with mitochondrial disease are more vulnerable to antitumor agents than healthy individuals. However, there have been no reports of chemotherapy in patients with mitochondrial disease or drug sensitivity studies in cells with mitochondrial dysfunction.

This report detailed a rare case of APL complicated by MELAS that was successfully treated with modified chemotherapy consisting of CA, ATRA, and reduced anthracycline. Anthracyclines exert antitumor effects through TOP2β inhibition and increased reactive oxygen species (ROS) production [3]. The increase in ROS is particularly closely associated with oxidative stress-related cardiac injury, which can often be fatal in patients with mitochondrial diseases [3, 11]. The patient achieved molecular remission promptly and maintained it despite the reduction in the key drug’s dosage. The clinical course suggests the vulnerability of cancer cells to anticancer drugs, which is possibly due to mitochondrial dysfunction. Additionally, an in vitro study revealed that the patient’s normal leukocytes presented significantly higher sensitivity to many drugs including mitoxantrone, than the healthy controls (Supplemental Fig. 1). Although our analyses did not include direct assessments of drug sensitivity in APL cells (a limitation of our study), mitochondrial dysfunction and oxidative stress may influence the effects of anticancer drugs to a greater extent than was previously believed, and these findings reflect that our treatment strategy involving the reduced chemotherapy dose was reasonable.

Recently, the association between oxidative stress and oncogenesis has attracted increasing attention. Warburg discovered that cancer cells produce ROS due to mutations in mtDNA and TCA cycle and/or electron transport chain dysfunction [20]. ROS play crucial roles in cancer development by causing oxidative DNA damage and genomic instability. In our patient, we cannot rule out the possibility that oxidative stress played a role in the development of APL. There are few reports of patients with mitochondrial diseases who developed malignancy [6–9], and it is unclear whether this is due to the rarity of mitochondrial diseases or their natural course, which can be fatal before old age [3]. However, with advances in the management and preventive medicine of mitochondrial diseases [21], the number of elderly patients with mitochondrial diseases is likely to increase, which may lead to an increased frequency of various types of cancers. Our experience suggests that chemotherapy for patients with mitochondrial diseases should be selected with caution.

Mitochondrial dysfunction may enhance drug sensitivity and therapeutic toxicity, to date this has not been confirmed. In this report, a patient with APL with complicating mitochondrial disease was successfully treated with reduced-concentration chemotherapy. Patients with mitochondrial diseases are more vulnerable to oxidative stress induced by anticancer drugs. Careful selection of a treatment approach for malignant tumors is thus necessary.

## Supplementary Information

Below is the link to the electronic supplementary material.

Supplementary file1 (TIF 97 KB) Supplemental Fig. 1 Average drug effect score (DES) in the patient and controls. We compared the average DES for each drug in the patient to that in the controls. Asterisk marks indicate a significant difference in drug sensitivity in the analysis of variance

Supplementary file2 (DOCX 19 KB)

Supplementary file3 (DOCX 20 KB)

Supplementary file4 (DOCX 43 KB)

## Data Availability

Raw data were generated at the Kanagawa Children’s Medical Center. The data supporting the findings of this study are available from the corresponding author upon request.
