# Supplementary figures and images for: A case of acute promyelocytic leukemia complicated by mitochondrial disease
Source: Int J Hematol. 2025 May 1;122(2):301–4. doi: 10.1007/s12185-025-03992-4 (PMC12304024; doi:10.1007/s12185-025-03992-4)

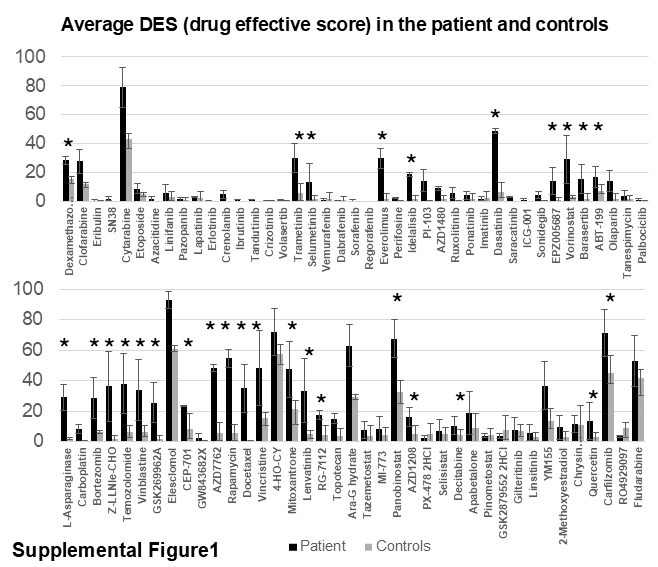

Supplement: Supplementary file 1 — Supplementary file1 (TIF 97 KB) Supplemental Fig. 1 Average drug effect score (DES) in the patient and controls. We compared the average DES for each drug in the patient to that in the controls. Asterisk marks indicate a significant difference in drug sensitivity in the analysis of variance [file 12185_2025_3992_MOESM1_ESM.tif]
