# Supplementary material for: A case of acute promyelocytic leukemia complicated by mitochondrial disease
Source: Int J Hematol. 2025 May 1;122(2):301–4. doi: 10.1007/s12185-025-03992-4 (PMC12304024; doi:10.1007/s12185-025-03992-4)
Supplement: Supplementary file 2 — Supplementary file2 (DOCX 19 KB) [file 12185_2025_3992_MOESM2_ESM.docx]

**Supplemental methods**

**<Drug sensitivity test>**

In the remission stage, peripheral blood samples were obtained from the patient at two different time points during maintenance therapy, on days when ATRA was not administered. Peripheral blood samples obtained from three healthy adults were used as controls. Mononuclear cells (MNC) were isolated and used for drug sensitivity testing (DST), as described previously [14, 15].

The sensitivity test included a drug panel routinely prepared in the laboratory, which included the drugs used in AML; 80 drugs of the panel and their final concentrations have been listed in supplemental table 1. For this assay, 10,000/10 μL MNC were seeded onto wells of a 384-well plate, each preloaded with 10 μL of culture medium containing 1 of tested drugs or their serial dilutions (5^–1^, 5^–2^, or 5^–3^). The culture medium without the drug was added to the control wells. After 4 days of culture, the viability of cells in each well was measured using the Cell Titer-Glo Luminescent Assay (Promega, Madison, WI, USA). To compare drug sensitivity among the samples, the drug effect score (DES) was used as previously reported [13, 14]; DES = 100 caused total cell death at all tested concentrations, whereas DES = 0 had no drug effect. Reference values for DES were obtained from peripheral blood mononuclear cells of healthy controls. The average DES and cell viability at each drug concentration were calculated for each of the two DSTs for the patient and the three examinations for the controls (Supplemental table 2). DESs were compared between patient and control samples for the drugs that showed efficacies of DES > 10.0 to either of the samples, and two-way analysis of variance was used for statistical comparisons. This study was approved by the relevant institutional ethics committee. The patient, his guardian, and healthy control volunteers provided written informed consent.

The samples from the patient showed significantly higher DES than those from the controls for 33 drugs (Supplemental figure 1).
