# Supplementary material for: A case of acute promyelocytic leukemia complicated by mitochondrial disease
Source: Int J Hematol. 2025 May 1;122(2):301–4. doi: 10.1007/s12185-025-03992-4 (PMC12304024; doi:10.1007/s12185-025-03992-4)
Supplement: Supplementary file 3 — Supplementary file3 (DOCX 20 KB) [file 12185_2025_3992_MOESM3_ESM.docx]

**Supplemental Table2：**A list of drugs and the final concentrations used in the drug-sensitivity test

| **Drug** | **Concentration** | **Drug** | **Concentration** |
| --- | --- | --- | --- |
| Dexamethasone | 100 µg/ml | ICG-001 | 1 µM |
| Clofarabine | 100 ng/ml | Sonidegib | 1 µM |
| Eribulin | 5 ng/ml | EPZ005687 | 1 µM |
| SN38 | 20 ng/ml | Vorinostat | 1 µM |
| Cytarabine | 100 µg/ml | Barasertib | 1 µM |
| Etoposide | 20 µg/ml | ABT-199 | 1 µM |
| Azacitidine | 1 µg/ml | Olaparib | 1 µM |
| Linifanib | 1 µM | Tanespimycin | 1 µM |
| Pazopanib | 1 µM | Palbociclib | 1 µM |
| Lapatinib | 1 µM | L-Asparaginase | 10 U/ml |
| Erlotinib | 1 µM | Carboplatin | 10 µg/ml |
| Crenolanib | 1 µM | Bortezomib | 50 ng/ml |
| Ibrutinib | 1 µM | Z-LLNle-CHO | 1 µM |
| Tandutinib | 1 µM | Temozolomide | 5 µg/ml |
| Crizotinib | 1 µM | Vinblastine | 10 ng/ml |
| Volasertib | 1 µM | GSK269962A | 1 µM |
| Trametinib | 1 µM | Elesclomol | 1 µM |
| Selumetinib | 1 µM | CEP-701 | 1 µM |
| Vemurafenib | 1 µM | GW843682X | 1 µM |
| Dabrafenib | 1 µM | AZD7762 | 1 µM |
| Sorafenib | 1 µM | Rapamycin | 1 µM |
| Regorafenib | 1 µM | Docetaxel | 1 µg/ml |
| Everolimus | 1 µM | Vincristine | 1 µg/ml |
| Perifosine | 1 µM | 4-HO-CY | 40 µg/ml |
| Idelalisib | 1 µM | Mitoxantrone | 500 ng/ml |
| PI-103 | 1 µM | Lenvatinib | 1 µM |
| AZD1480 | 1 µM | RG-7112 | 1 µM |
| Ruxolitinib | 1 µM | Topotecan | 500 ng/ml |
| Ponatinib | 1 µM | Ara-G hydrate | 88.5 uM |
| Imatinib | 1 µM | Tazemetostat | 1 uM |
| Dasatinib | 1 µM | MI-773 (SAR405838) | 1 uM |
| Saracatinib | 1 µM | Panobinostat | 1 uM |
| Ara-G hydrate | 88.5 uM | GSK2879552 2HCl | 1 uM |
| Tazemetostat | 1 uM | Gilteritinib (ASP2215) | 1 uM |
| MI-773 (SAR405838) | 1 uM | Linsitinib (OSI-906) | 1 uM |
| Panobinostat | 1 uM | YM155 | 1 uM |
| AZD1208 | 1 uM | 2-Methoxyestradiol | 1 uM |
| PX-478 2HCl | 1 uM | Chrysin Dimethylether | 10 ug/ml |
| Selisistat (EX 527) | 1 uM | Quercetin | 1 uM |
| Decitabine | 1 uM | Carfilzomib (PR-171) | 1 uM |
| Apabetalone | 1 uM | RO4929097 | 1 uM |
| Pinometostat | 1 uM | Fludarabine | 5 ug/ml |
