# Supplementary material for: A case of acute promyelocytic leukemia complicated by mitochondrial disease
Source: Int J Hematol. 2025 May 1;122(2):301–4. doi: 10.1007/s12185-025-03992-4 (PMC12304024; doi:10.1007/s12185-025-03992-4)
Supplement: Supplementary file 4 — Supplementary file4 (DOCX 43 KB) [file 12185_2025_3992_MOESM4_ESM.docx]

Supplemental table 1: average cell viability in each drug concentrations and DES

| **drugs** | **Patient** | | | | | **Controls** | | | | | **p-value** |
| --- | --- | --- | --- | --- | --- | --- | --- | --- | --- | --- | --- |
|  | **×1/125** | **×1/25** | **×**  **1/5** | **×**  **1** | **DES** | **×1/125** | **×1/25** | **×**  **1/5** | **×**  **1** | **DES** |  |
| **dexamthesone** | **0.764** | **0.737** | **0.692** | **0.451** | **28.4** | **0.869** | **0.879** | **0.798** | **0.705** | **15.4** | **0.023** |
| **clofarabine** | **1.13** | **0.628** | **0.436** | **0.244** | **27.7** | **1.09** | **0.963** | **0.590** | **0.490** | **12.1** | **0.120** |
| **eribulin** | **1.14** | **1.17** | **1.07** | **1.16** | **0.570** | **1.09** | **1.10** | **1.09** | **1.08** | **0.197** | **0.167** |
| **SN38** | **1.12** | **1.05** | **1.14** | **0.869** | **1.85** | **1.10** | **1.12** | **1.14** | **1.08** | **0.000** | **0.298** |
| **cytarabine** | **0.282** | **0.154** | **0.158** | **0.150** | **78.8** | **0.809** | **0.429** | **0.286** | **0.289** | **43.4** | **0.063** |
| **etoposide** | **1.16** | **0.976** | **0.787** | **0.468** | **8.94** | **1.10** | **1.08** | **0.937** | **0.576** | **5.16** | **0.195** |
| **azacitidine** | **1.08** | **1.03** | **1.15** | **1.15** | **1.88** | **1.05** | **1.11** | **1.04** | **1.09** | **0.347** | **0.505** |
| **Linifanib** | **1.01** | **1.07** | **1.10** | **1.38** | **6.03** | **0.990** | **1.00** | **0.946** | **0.964** | **3.13** | **0.155** |
| **pazopanib** | **1.00** | **1.17** | **1.09** | **0.947** | **1.62** | **1.04** | **1.09** | **1.02** | **0.939** | **1.09** | **0.326** |
| **Lapatinib** | **0.959** | **1.02** | **1.05** | **1.05** | **3.10** | **1.01** | **1.00** | **0.988** | **1.01** | **2.32** | **0.525** |
| **erlotinib** | **1.14** | **1.05** | **1.14** | **1.06** | **0.215** | **1.09** | **1.10** | **1.08** | **1.11** | **0.000** | **0.973** |
| **crenolanib** | **1.15** | **1.07** | **1.07** | **0.678** | **4.96** | **1.11** | **1.11** | **1.04** | **1.05** | **0.000** | **0.442** |
| **ibrutinib** | **1.10** | **1.12** | **1.12** | **1.14** | **0.755** | **1.08** | **1.10** | **1.04** | **1.16** | **0.0900** | **0.315** |
| **tandutinib** | **1.13** | **1.09** | **1.16** | **0.979** | **0.820** | **1.11** | **1.12** | **1.10** | **1.10** | **0.000** | **0.710** |
| **crizotinib** | **1.12** | **1.18** | **0.975** | **1.02** | **0.465** | **1.14** | **1.08** | **1.05** | **1.10** | **0.313** | **0.651** |
| **volasertib** | **1.14** | **1.04** | **1.03** | **0.909** | **0.900** | **1.09** | **1.07** | **1.14** | **1.11** | **0.300** | **0.271** |
| **trametinib** | **0.755** | **0.678** | **0.610** | **0.636** | **30.2** | **0.930** | **0.983** | **0.92** | **0.986** | **5.97** | **0.005** |
| **selumetinib** | **0.963** | **0.903** | **0.891** | **0.761** | **13.5** | **1.08** | **1.02** | **0.986** | **0.985** | **1.60** | **0.017** |
| **vemurafenib** | **1.14** | **1.03** | **1.06** | **1.04** | **1.00** | **1.18** | **0.999** | **1.04** | **0.985** | **1.97** | **0.458** |
| **dabrafenib** | **1.09** | **1.01** | **1.13** | **1.10** | **0.840** | **1.02** | **1.01** | **1.01** | **1.00** | **1.23** | **0.068** |
| **sorafenib** | **1.29** | **1.09** | **1.24** | **1.14** | **0.630** | **1.12** | **1.09** | **1.09** | **1.13** | **0.000** | **0.168** |
| **regorafenib** | **1.03** | **1.08** | **1.14** | **1.17** | **0.000** | **1.08** | **1.09** | **1.17** | **1.12** | **0.000** | **0.680** |
| **everolimus** | **0.746** | **0.690** | **0.635** | **0.643** | **29.7** | **0.997** | **1.02** | **1.00** | **1.02** | **1.77** | **0.001** |
| **perifosine** | **1.03** | **0.943** | **1.07** | **0.895** | **2.70** | **1.12** | **1.10** | **1.12** | **1.11** | **0.207** | **0.039** |
| **idelalisib** | **0.843** | **0.826** | **0.777** | **0.691** | **18.7** | **1.05** | **1.05** | **1.02** | **1.01** | **1.45** | **0.002** |
| **PI-103** | **0.972** | **0.893** | **0.702** | **0.440** | **14.3** | **1.09** | **1.06** | **1.06** | **1.05** | **0.407** | **0.068** |
| **AZD1480** | **0.979** | **0.933** | **0.809** | **0.684** | **9.03** | **1.03** | **1.04** | **1.01** | **1.05** | **1.52** | **0.082** |
| **ruxolitinib** | **1.01** | **0.993** | **0.890** | **0.846** | **5.68** | **1.06** | **1.04** | **1.08** | **1.03** | **0.267** | **0.058** |
| **ponatinib** | **1.08** | **0.977** | **0.930** | **0.770** | **4.29** | **1.04** | **0.988** | **1.03** | **1.24** | **1.85** | **0.324** |
| **imatinib** | **1.00** | **1.00** | **1.15** | **0.939** | **1.86** | **1.01** | **1.00** | **0.984** | **0.989** | **2.19** | **0.637** |
| **dasatinib** | **0.526** | **0.483** | **0.529** | **0.506** | **48.9** | **0.935** | **0.953** | **0.912** | **0.929** | **6.35** | **<0.001** |
| **saracatinib** | **1.12** | **1.00** | **1.07** | **0.704** | **3.23** | **1.12** | **1.09** | **1.11** | **1.05** | **0.000** | **0.230** |
| **ICG-001** | **1.11** | **1.01** | **1.07** | **0.953** | **1.27** | **1.08** | **1.07** | **1.06** | **1.07** | **0.000** | **0.381** |
| **Sonidegib** | **0.985** | **1.00** | **0.986** | **0.858** | **4.28** | **1.22** | **1.13** | **1.08** | **1.08** | **0.247** | **0.016** |
| **EPZ005687** | **0.981** | **0.825** | **0.759** | **0.711** | **14.3** | **1.07** | **1.06** | **1.08** | **1.03** | **0.860** | **0.022** |
| **vorinostat** | **0.720** | **0.832** | **0.713** | **0.265** | **29.0** | **1.09** | **1.05** | **0.984** | **0.731** | **3.01** | **0.009** |
| **barasertib** | **0.847** | **0.916** | **0.747** | **0.757** | **15.6** | **1.03** | **1.06** | **0.991** | **1.02** | **2.00** | **0.005** |
| **ABT-199** | **0.909** | **0.899** | **0.681** | **0.497** | **16.7** | **1.03** | **1.00** | **0.890** | **0.544** | **7.59** | **0.037** |
| **Olaparib** | **0.847** | **0.973** | **0.731** | **0.799** | **13.9** | **1.02** | **0.991** | **1.03** | **0.980** | **1.92** | **0.063** |
| **tanespimycin** | **1.05** | **0.989** | **1.01** | **0.903** | **4.14** | **1.05** | **1.08** | **0.995** | **0.948** | **1.66** | **0.282** |
| **Palbociclib** | **1.16** | **1.04** | **1.11** | **0.944** | **1.12** | **1.04** | **1.05** | **1.04** | **1.02** | **0.127** | **0.587** |
| **L-Asparaginase** | **0.912** | **0.656** | **0.418** | **0.377** | **29.0** | **1.18** | **1.02** | **0.965** | **0.877** | **1.73** | **0.007** |
| **carboplatin** | **1.12** | **0.937** | **0.884** | **0.525** | **8.11** | **1.08** | **1.03** | **1.24** | **0.969** | **0.300** | **0.157** |
| **Bortezomib** | **0.791** | **0.834** | **0.592** | **0.140** | **28.7** | **1.06** | **1.19** | **1.03** | **0.348** | **6.28** | **0.008** |
| **Z-LLNle-CHO** | **0.635** | **0.634** | **0.666** | **0.604** | **36.3** | **1.02** | **1.05** | **0.993** | **1.02** | **1.91** | **<0.001** |
| **Temozolomide** | **0.663** | **0.613** | **0.549** | **0.550** | **38.0** | **0.967** | **0.933** | **0.881** | **0.864** | **6.61** | **<0.001** |
| **Vinblastine** | **0.685** | **0.663** | **0.661** | **0.545** | **33.8** | **0.939** | **0.974** | **0.851** | **0.948** | **6.52** | **0.008** |
| **GSK269962A** | **0.715** | **0.856** | **0.661** | **0.664** | **25.5** | **1.10** | **1.00** | **0.995** | **1.00** | **1.46** | **0.010** |
| **Elesclomol** | **0.143** | **<0.01** | **<0.01** | **0.015** | **92.9** | **0.795** | **0.134** | **0.0262** | **0.066** | **60.5** | **0.237** |
| **CEP-701** | **0.861** | **0.709** | **0.693** | **0.566** | **23.8** | **0.935** | **0.927** | **0.878** | **0.869** | **8.23** | **0.026** |
| **GW843682X** | **1.04** | **1.01** | **0.992** | **1.05** | **2.62** | **1.07** | **1.02** | **1.15** | **1.06** | **0.343** | **0.270** |
| **AZD7762** | **0.578** | **0.444** | **0.490** | **0.507** | **48.3** | **0.982** | **0.941** | **0.954** | **0.909** | **5.37** | **<0.001** |
| **Rapamycin** | **0.471** | **0.424** | **0.468** | **0.439** | **54.6** | **0.927** | **0.965** | **0.940** | **0.946** | **5.84** | **<0.001** |
| **Docetaxel** | **0.708** | **0.551** | **0.745** | **0.544** | **34.9** | **1.10** | **1.01** | **1.07** | **0.973** | **0.390** | **<0.001** |
| **vincristine** | **0.563** | **0.489** | **0.527** | **0.347** | **48.5** | **0.876** | **0.814** | **0.877** | **0.727** | **15.7** | **<0.001** |
| **4-HO-CY** | **0.434** | **0.278** | **0.019** | **0.020** | **71.8** | **0.663** | **0.402** | **0.0313** | **0.0228** | **57.1** | **0.176** |
| **Mitoxantrone** | **0.515** | **0.594** | **0.447** | **0.488** | **47.4** | **0.757** | **0.877** | **0.663** | **0.920** | **21.1** | **0.009** |
| **lenvatinib** | **0.825** | **0.737** | **0.549** | **0.400** | **33.5** | **0.953** | **1.03** | **0.947** | **0.834** | **5.02** | **0.020** |
| **RG-7112** | **0.785** | **0.909** | **0.793** | **0.817** | **17.4** | **0.972** | **0.965** | **0.983** | **0.937** | **4.23** | **0.022** |
| **topotecan** | **1.06** | **0.838** | **0.873** | **0.357** | **15.0** | **1.00** | **1.01** | **0.971** | **0.881** | **3.92** | **0.235** |
| **Ara-G hydrate** | **0.543** | **0.278** | **0.170** | **0.160** | **62.9** | **1.03** | **0.641** | **0.360** | **0.246** | **29.3** | **0.051** |
| **Tazemetostat** | **0.943** | **0.885** | **0.993** | **0.981** | **7.74** | **1.02** | **0.999** | **1.15** | **0.991** | **3.95** | **0.061** |
| **MI-773** | **0.893** | **0.985** | **0.937** | **0.905** | **8.40** | **0.939** | **1.16** | **0.935** | **1.32** | **4.04** | **0.263** |
| **Panobinostat** | **0.458** | **0.264** | **0.166** | **0.132** | **67.6** | **0.913** | **0.628** | **0.437** | **0.304** | **32.3** | **0.014** |
| **AZD1208** | **0.846** | **0.858** | **0.797** | **0.826** | **16.0** | **1.09** | **0.962** | **0.907** | **0.927** | **4.70** | **0.028** |
| **PX-478 2HCl** | **1.05** | **0.991** | **0.989** | **1.03** | **2.12** | **0.997** | **0.983** | **0.972** | **0.966** | **4.70** | **0.082** |
| **Selisistat** | **0.935** | **0.936** | **0.970** | **0.897** | **7.25** | **1.04** | **1.06** | **1.12** | **1.02** | **5.14** | **0.001** |
| **Decitabine** | **0.921** | **0.937** | **0.860** | **0.702** | **10.4** | **1.28** | **1.14** | **1.01** | **0.999** | **4.19** | **0.012** |
| **Apabetalone** | **0.777** | **0.865** | **0.823** | **0.849** | **18.9** | **1.24** | **1.01** | **1.23** | **0.974** | **8.61** | **0.048** |
| **Pinometostat** | **0.934** | **1.06** | **0.968** | **0.973** | **3.98** | **1.02** | **0.926** | **1.07** | **0.965** | **4.20** | **0.893** |
| **GSK2879552 2HCl** | **0.994** | **0.909** | **1.01** | **1.42** | **3.55** | **0.961** | **0.933** | **0.999** | **0.896** | **7.55** | **0.369** |
| **Gilteritinib** | **0.936** | **1.03** | **0.912** | **0.764** | **7.80** | **0.990** | **0.932** | **0.927** | **0.748** | **7.09** | **0.759** |
| **Linsitinib** | **0.972** | **1.07** | **1.00** | **0.832** | **5.88** | **1.17** | **1.12** | **1.02** | **0.881** | **3.14** | **0.137** |
| **YM155** | **0.805** | **0.762** | **0.232** | **0.0789** | **36.7** | **0.909** | **0.978** | **0.806** | **0.441** | **13.7** | **0.053** |
| **2-Methoxyestradiol** | **0.959** | **0.865** | **0.933** | **0.899** | **9.36** | **0.984** | **1.07** | **0.977** | **1.211** | **3.06** | **0.123** |
| **ChrysinDimethylether** | **0.917** | **1.02** | **0.928** | **0.383** | **11.7** | **0.967** | **0.958** | **1.06** | **0.827** | **10.8** | **0.282** |
| **Quercetin** | **1.02** | **0.871** | **0.898** | **0.832** | **13.6** | **1.14** | **1.02** | **1.25** | **1.20** | **3.16** | **0.035** |
| **Carfilzomib** | **0.584** | **0.039** | **0.073** | **0.024** | **71.0** | **0.951** | **0.336** | **0.363** | **0.0889** | **44.7** | **0.030** |
| **RO4929097** | **0.956** | **1.04** | **1.02** | **0.975** | **3.51** | **0.930** | **0.884** | **0.929** | **0.911** | **8.77** | **0.057** |
| **fludarabine** | **0.682** | **0.392** | **0.202** | **0.165** | **52.6** | **0.892** | **0.424** | **0.240** | **0.178** | **41.5** | **0.209** |
